# Let the Shoemaker Stick to His Last

**Published:** 1897-12

**Authors:** 


### LET THE SHOEMAKER STICK TO HIS LAST.

The following note is taken from the current number of a prominent agricultural paper :

" A special inquiry concerning abortion and milk-fever is also being made, and the probabilities are that a bulletin of information on this subject will be issued in a short time."

This refers to the Agricultural Experiment Station in New Jersey. This Experiment Station is one of the few in the United States that has no veterinarian attached to its staff, although for several years past it has issued bulletins on veterinary subjects. These have been prepared by a gentleman who is a very good biologist, but has neither practical nor theoretical knowledge of the diseases of animals. When such authors write on unfamiliar subjects their publications may be of value if they are confined to compilations from standard authorities; but when they attempt to voice their own opinions their so-called information is frequently absurd and misleading to an extreme degree.

*Do Experiment Stations ask horticulturists to prepare bulletins on butter-making ? Do they ask dairymen to conduct experiments on the testing of seeds ? Are irrigation-engineers supposed to have expert knowledge on the rearing of calves, and are entomologists ever called upon to write observations upon the construction of silos ?* If not, why should a biologist be obliged to prepare bulletins on tuberculosis, milk-fever, and abortion ? Agricultural science and the work of Experiment Stations are now specialized to such a degree that it is not only absurd but wrong for any but acknowledged experts to prepare bulletins upon important subjects in the line of such work.

Veterinary science deserves to rank as an independent, well-developed specialty. It has schools, professors, and a large, intelligent, active, progressive body of men, devoting all of their time to its development. It has also an immense literature. For all of this to be disregarded by the New Jersey Experiment Station is a rank injustice to the farmers of that State.

*If bulletins on veterinary subjects are important enough to justify*

*their publication by an Experiment Station, they are certainly of sufficient importance to justify the employment of an expert to prepare them.*

